# Specimens of Dental Pathology, Gutta Percha, &c

**Published:** 1856-06

**Authors:** Alexander M. Holmes

**Affiliations:** Morrisville, Madison County, N. York.


*Specimens of Dental Pathology, Gutta Percha, &c.* By ALEXANDER M. HOLMES, D. D. S., Morrisville, Madison county, N. York.

Inclosed I send you a molar tooth the ossified pulp of which completely filled the cavity at the time it was removed. This tooth had been filled, and the nerve protected by a metallic cap covered with gutta percha. One year afterwards the tooth was extracted preparatory to inserting an artificial denture.

I send also a few specimens of colored gutta percha; altho' they by no means represent the extent and variety of shading of which a pure article of this gum is susceptible, these having been prepared from a very dark and impure specimen.

As gutta percha has to some extent engaged the attention of the profession, I take pleasure in communicating to you the result of my experience in its use, for temporary purposes during the past five years.

---

*Cœluis Aurelinus de Morbis atcutis aque chronicis. Amstel. 1709. 4to.

As a temporary filling, in cases where the lining membrane of the pulp cavity is nearly or quite exposed, and the dentine in an inflamed condition, I regard the preparation of Dr. Hill as superior to any material with which I am acquainted. Filling with this article does not necessarily increase the irritation; it is easily accomplished, and possesses the two-fold advantage of a non-conductor, and of preserving the tooth until its healthy condition is so far restored as to admit of a gold filling. Many teeth may thus be saved with their nervous tissues unimpaired, which, had no preparatory treatment been adopted, must either have been lost or the patient subjected to the unpleasant operation of destroying the nerve.

As a base for the support of artificial teeth, gutta percha can be advantageously used; especially in temporary cases, as when the gums and alveoli are in a diseased condition, and the irritation of a metallic plate would render its use impracticable.

Teeth constructed on a base of this material can be worn by the patient with comparative ease. It is also admirably adapted to fulfil the indication frequently met with in persons of advanced age, who, by reason of the tenderness of their gums, cannot be persuaded to endure the irritation resulting from the first use of a metallic plate.

Again, where (as in many cases), we desire to restore the *contour* of the face, it may be neatly and effectually done by covering the plate, properly extended, with this material. To accomplish this, the extended portion of the plate to which the gutta percha is to be applied, should be thickly perforated in order to allow the gum to pass through and unite the two layers, thereby increasing its durability and preventing its pulling from the plate. I have usually made these punctures with the punch in ordinary use for lining plate teeth.

It will also be found very useful in the laboratory when the ordinary impression cup cannot be used advantageously, as one can be formed of this material in a few minutes, and of any required shape.

The process I have adopted in the preparation of this article as a support for artificial teeth is very simple. Where it is desirable to give it the color of the natural gums, a pure article of gutta percha is essential to success. No amount of coloring will perfectly obviate the dead shade imparted to the gum by the impurities contained in most of the specimens found in our markets. The coloring matter having a vegetable origin, and being minute in quantity, does not impart to the gum any injurious property, or in the least impair its texture.

My process is this: Take any quantity of chloroform suitably colored to produce the desired shade of body, and add to it as much of the gum as it will dissolve; having done this and shaken it well, the solution may be poured out upon a marble slab to allow the chloroform to evaporate, after which the remaining mass should be softened in hot water and well worked together. It may then be pressed, rolled or drawn out into any required shape.

A suitable variety of shades may be given it by the use of lakes, varying from purple to pink, adding, at the same time a small quantity of gum gamboge. If the gutta percha has originally too dark a shade, a little white may be required. The coloring material should be finely pulverized, and ground in alcohol before it is added to the chloroform.

According to my experience, the best method of using gutta percha as a support for artificial teeth, is to swage a narrow plate, perforate it as above directed and to this attach the teeth; or if a clasp case, then the clasps also, in the usual way.

After the base has been suitably fitted to the die, the plate and teeth should be heated and pressed down firmly upon the gutta percha, which, passing through this "cribriform plate," and uniting with that on the opposite surface, thus render the work firm and durable. If an atmospheric case, it should be supported by a narrow strip of plate, or two or more pieces of wire extended across the roof of the mouth, or if preferred, a wire frame like that recommended by Dr. Trueman may be used. The former process has, however, been most satisfactory in my practice as in most cases before the absorption of the alveoli, the teeth used must necessarily be short, thereby rendering their attachment by any other method I have yet tried, very difficult and unsatisfactory.

Without some such support as that above mentioned, a gutta percha case of sufficient strength to answer the purpose will be found too bulky and cumbersome to be worn by our patients.

The piece should be kept dry until a sufficient quantity of the gum has been moulded upon it, in order that the union may be perfect, after which, in the finishing, either cold or hot water may be used, as best suits the convenience of the operator.

That the above hastily described process may yet be greatly improved, I shall not permit myself to doubt; and the suggestions I have ventured to make have been less with a view to instruct the members of our enlightened profession, than to enlist them in the further investigation of this subject.—*American Journal of Dental Science.*

